# Selection and exploitation of prevalent, tandemly repeated genomic targets for improved real-time PCR-based detection of *Wuchereria bancrofti* and *Plasmodium falciparum* in mosquitoes

**DOI:** 10.1371/journal.pone.0232325

**Published:** 2020-05-01

**Authors:** Michael F. Zulch, Nils Pilotte, Jessica R. Grant, Corrado Minetti, Lisa J. Reimer, Steven A. Williams

**Affiliations:** 1 Department of Biological Sciences, Smith College, Northampton, Massachusetts, United States of America; 2 Molecular and Cellular Biology Program, University of Massachusetts, Amherst, Massachusetts, United States of America; 3 Department of Vector Biology, Liverpool School of Tropical Medicine, Liverpool, United Kingdom; Instituto Rene Rachou, BRAZIL

## Abstract

**Background:**

Optimization of polymerase chain reaction (PCR)-based diagnostics requires the careful selection of molecular targets that are both highly repetitive and pathogen-specific. Advances in both next-generation sequencing (NGS) technologies and bioinformatics-based analysis tools are facilitating this selection process, informing target choices and reducing labor. Once developed, such assays provide disease control and elimination programs with an additional set of tools capable of evaluating and monitoring intervention successes. The importance of such tools is heightened as intervention efforts approach their endpoints, as accurate and complete information is an essential component of the informed decision-making process. As global efforts for the control and elimination of both lymphatic filariasis and malaria continue to make significant gains, the benefits of diagnostics with improved analytical and clinical/field-based sensitivities and specificities will become increasingly apparent.

**Methodology/Principal findings:**

Coupling Illumina-based NGS with informatics approaches, we have successfully identified the tandemly repeated elements in both the *Wuchereria bancrofti* and *Plasmodium falciparum* genomes of putatively greatest copy number. Utilizing these sequences as quantitative real-time PCR (qPCR)-based targets, we have developed assays capable of exploiting the most abundant tandem repeats for both organisms. For the detection of *P*. *falciparum*, analysis and development resulted in an assay with improved analytical and field-based sensitivity vs. an established ribosomal sequence-targeting assay. Surprisingly, analysis of the *W*. *bancrofti* genome predicted a ribosomal sequence to be the genome’s most abundant tandem repeat. While resulting cycle quantification values comparing a qPCR assay targeting this ribosomal sequence and a commonly targeted repetitive DNA sequence from the literature supported our finding that this ribosomal sequence was the most prevalent tandemly repeated target in the *W*. *bancrofti* genome, the resulting assay did not significantly improve detection sensitivity in conjunction with field sample testing.

**Conclusions/Significance:**

Examination of pathogen genomes facilitates the development of PCR-based diagnostics targeting the most abundant and specific genomic elements. While in some instances currently available tools may deliver equal or superior performance, systematic analysis of potential targets provides confidence that the selected assays represent the most advantageous options available and that informed assay selection is occurring in the context of a particular study’s objectives.

## Introduction

Human malaria and lymphatic filariasis (LF), are mosquito-transmitted tropical diseases that disproportionately affect economically disadvantaged nations. Due to their public health impact and resulting economic burden, elimination efforts for these diseases continue to expand with assistance from large-scale collaborative operations such as the Global Malaria Programme [1–2] and the Global Programme to Eliminate Lymphatic Filariasis (GPELF) [3]. Thanks to such coordinated undertakings, significant strides are being made to reduce the incidences of both diseases. However, as the trend towards elimination continues, and disease incidence declines, accurate programmatic decision-making will require the development of new and improved diagnostic tools with the capacity to reliably assess infection status and measure intervention success.

Currently, World Health Organization (WHO)-recommended methods for the detection of both malaria and lymphatic filariasis (LF) rely upon either the microscopic examination of blood samples or serological antigen/antibody testing [4–6]. While widely used and important for programmatic decision making processes [7–9], these approaches are dependent upon human blood sampling and considerable evidence exists demonstrating the potential for these testing methods to lead to both false-positive and false-negative results [6, 10–12]. While requiring more advanced infrastructure, DNA-based assays utilizing quantitative real-time polymerase chain reaction (qPCR), are able to improve upon diagnostic sensitivity and specificity of detection for these diseases [6, 13–14]. PCR-based assays also enable the testing of sample types other than blood. Most importantly, in the context of LF and malaria, this capacity allows researchers to indirectly and non-invasively sample a population using unique methods such as molecular xenomonitoring (MX): the testing of hematophagous arthropods for the presence of parasite-derived genetic material [15–18].

While PCR-based assays targeting the causative agents of LF and human malaria have existed for over two decades [19–20], recent advances in genome biology, next-generation sequencing (NGS), and bioinformatics are facilitating increasingly systematic approaches to assay design [21]. Through the computational analysis of whole genome sequencing data, highly repetitive, species-specific, tandemly repeated DNA elements can be readily and rapidly identified [22–23]. These sequences can then be utilized as PCR targets, resulting in significant improvements to both analytical and clinical/field sensitivity and specificity of detection when compared with less prevalent, more conserved regions that have traditionally been employed [21, 24–26]. Under experimental conditions, such targets result in reduced cycle quantification (Cq) values for a given concentration of template DNA. Of greater diagnostic relevance, repeat-based assays have the potential to lower an assay’s detection threshold as the increased representation of the targets within the genome improves the probability that a given fragment of DNA may contain a target sequence. This improves the robustness of such an assay, helping to facilitate detection from impure or degraded samples, as well as samples resulting from sub-optimal extractions. Improvements to robustness are particularly pronounced when assay targets are tandemly repeated, as random shearing or degradation of DNA is likely to leave a greater number of target regions intact. As such, the utilization of highly repetitive, tandemly repeated targets generates improved confidence in results due to greater diagnostic sensitivity. With intervention efforts continuing to reduce infection frequency and parasite burden, the importance of assay sensitivity and reliability has been magnified, making such detection methods increasingly critical to successful programmatic efforts.

In an attempt to improve upon the analytical and field sensitivities of PCR-based pathogen detection for the causative agents of LF and human malaria, we here describe the development of two novel, TaqMan-based, qPCR assays targeting *Wuchereria bancrofti* and *Plasmodium falciparum*. Utilizing next-generation sequencing, the most highly repetitive tandemly repeated targets within each genome were identified. Index assays targeting these high copy-number DNA sequences were then designed, optimized, and validated against existing reference qPCR assays using field-collected samples. Comparison with these existing qPCR assays supported our target copy-number predictions, as Cq values were reduced when targeting the newly identified repeat elements from both organisms. Primarily for *P*. *falciparum* detection, these reductions in Cq value translated to improved field sensitivity, reducing false negative results through the detection of an increased number of positive samples.

## Materials and methods

### Next-generation sequencing and repeat DNA analysis

In preparation for next-generation sequencing (NGS), *W*. *bancrofti* var. Pacifica genomic DNA was isolated from microfilariae originally obtained from a Polynesian donor [27]. Genomic DNA from *Plasmodium falciparum*, Strain W2, MRA-157G was provided by BEI Resources. For each pathogen, library preparations for NGS were performed using the Nextera DNA Library Prep Kit (Illumina, San Diego, CA) in accordance with the manufacturer’s suggested protocol. Paired-end sequencing of the prepared libraries occurred on the MiSeq instrument (Illumina) using a v3 150-cycle reagent kit (Illumina) for *W*. *bancrofti* and a v2 300-cycle kit (Illumina) for *P*. *falciparum*. Sequence reads were analyzed using default parameters for both RepeatExplorer2 [22] and Tandem Repeat Analyzer (TAREAN) [23], Galaxy-based tools designed to identify repetitive DNA elements and satellite DNA sequences from paired-end whole genome sequencing data (publically available at galaxy-elixir.cerit-sc.cz). While we have previously described the use of RepeatExplorer for assay development purposes [24–26], TAREAN builds upon the algorithms developed for RepeatExplorer. Whereas, RepeatExplorer identifies and bins NGS sequence reads into clusters based on similarity, and then ranks those clusters based on the number of reads binned [22], TAREAN identifies clusters that are predicted to have a tandem arrangement within the genome and builds consensus sequences for these clusters. These predictions of tandem arrangement are based upon the identities and abundances of DNA sequences that flank elements mapping to the consensus [23]. The output of TAREAN is a list of putative tandemly repeated sequences built from each predicted cluster. From the output generated from both RepeatExplorer2 and TAREAN, targets for each qPCR assay were chosen.

### Assay design

#### Target selection

Utilizing consensus sequences, monomer lengths, and putative estimates of genomic proportions resulting from RepeatExplorer2 and TAREAN analyses, a candidate target from each pathogen was selected for further validation. For both *W*. *bancrofti* and *P*. *falciparum*, these candidate targets were predicted to be the genomic elements of greatest copy number having tandem arrangement and sufficient length to facilitate the design of a qPCR assay. Utilizing default parameters of the PrimerQuest Tool (Integrated DNA Technologies, Coralville, IA) TaqMan-based assays (hereafter referred to as the *W*. *bancrofti* Tandem Repeat #1 [*Wb* TR1] and *P*. *falciparum* Tanderm Repeat #1 [*Pf* TR1] assays) were designed for both targets, and a forward primer, reverse primer, and double-quenched 6FAM-ZEN/3IABkFQ probe (Integrated DNA Technologies) were synthesized.

#### Primer optimization

For each assay, optimal forward and reverse primer concentrations were determined using a previously described concentration matrix [24]. Briefly, five concentrations of forward and reverse primers (1 μM, 500 nM, 250 nM, 125 nM, 62.5 nM) were tested in every possible pairing, resulting in a 5 x 5 matrix testing 25 experimental combinations. All testing was performed in duplicate 10 μL reactions containing 5 μL of TaqPath ProAmp Master Mix (ThermoFisher Scientific, Waltham, MA), 2 μL of primer mix diluted to each appropriate experimental concentration, and 250 nM probe. Reactions were performed using the StepOnePlus Real-Time PCR System (ThermoFisher Scientific). Cycling conditions for both assays consisted of an initial hold at 50°C for 2 minutes, followed by a 10 minute incubation at 95°C. Following incubation, 40 cycles of 95°C for 15 seconds and 60°C for 1 minute were performed to allow for denaturation of template and annealing/extension of primers respectively. Optimal primer concentrations for each assay were determined to be the paired concentrations which produced the lowest mean Cq values.

#### Validation of analytical specificity

*In-silico* specificity was analyzed for each assay using Primer-BLAST, a primer alignment software from the National Center for Biotechnology Information (NCBI). All alignments were performed against NCBI’s RefSeq representative genomes database. Subsequent experimental validation was then performed by testing against a panel of genomic DNA isolates from both closely and more distantly related pathogens as well as human DNA. Finally, to ensure that each assay would successfully allow for the amplification of the intended target from geographically distinct isolates of pathogen, testing of eight different DNA extracts of *P*. *falciparum* genomic DNA obtained from BEI Resources (www.beiresources.org) was performed, as was the testing of *W*. *bancrofti-*positive DNA extracts from mosquito samples originating from six independent locations (American Samoa, French Polynesia, Ghana, Haiti, Sri Lanka, Tuvalu, and Zanzibar). All validation reactions were performed in duplicate using the optimized assay conditions and the cycling protocol described above.

#### Template titration

To evaluate the reaction efficiencies and analytical sensitivities of our newly described qPCR assays and to compare them with currently employed qPCR assays [16, 28], template limiting experiments were performed. Sequences and references for “long DNA repeat” (LDR) sequence-targeting (*W*. *bancrofti* detection) and ribosomal sequence-targeting (*P*. *falciparum*) primer-probe pairings used during this testing are found in Table 1. For each assay, 1:10 serial dilutions of genomic DNA were created, generating a panel of standards with concentrations ranging from 0.5 ng/μL to 50 ag/μL. Dilution panels were then tested, with each concentration undergoing analysis in triplicate 10 μL reactions. These reactions were performed using 2 μL of template and with the primer concentrations determined to be optimal for each assay. Each assay’s reaction efficiency was determined using the mean Cq values resulting from analysis of the five reactions with the highest input amount of DNA (1 ng, 100 pg, 10 pg, 1 pg, and 100 fg). Subsequently, for each pathogen, the lowest concentration of template at which both assays undergoing comparison produced three replicate positive reactions was identified. This concentration was then used to generate a second dilution series consisting of eight doubling dilutions. Eight replicates of each dilution within this second series were then tested with both our new and the existing qPCR assays, and comparative analytical sensitivities were evaluated based on the percentage of positive results obtained.

**Table 1:** Primer/probe sequences and reference information for LDR-targeting and ribosomal sequence-targeting assays used for comparative testing with index assays.

***Wuchereria bancrofti***	**5’ → 3’ Orientation**	**Relevant Reference**
Forward Primer	ATTTTGATCATCTGGGAACGTTAATA	Rao et al, 2006 [16]
Reverse Primer	CGACTGTCTAATCCATTCAGAGTGA	
Probe	ATCTGCCCATAGAAATAACTA	
***Plasmodium falciparum***	**5’ → 3’ Orientation**	**Relevant Reference**
Forward Primer	ATTGCTTTTGAGAGGTTTTGTTACTTT	Kamau et al, 2013 [28]
Reverse Primer	GCTGTAGTATTCAAACACAATGAACTCAA	
Probe	CATAACAGACGGGTAGTCAT	

#### Assay validation using field samples

To evaluate the field sensitivity and specificity of our new qPCR assays, each assay was tested using a set of DNA extracts isolated from field-collected mosquito pools gathered as part of two unrelated studies. For the detection of *W*. *bancrofti*, 436 samples collected from Haiti (n = 238) and Zanzibar (n = 198) were tested in duplicate reactions utilizing the optimized primer/probe concentrations described above. The results for each sample were compared to results obtained using the previously described LDR qPCR assay run in accordance with published conditions [16]. For the detection of *P*. *falciparum*, comparative testing of 616 samples, collected in Ghana, was performed using both our newly described index assay and a previously published reference assay targeting the 18S ribosomal sequence [28]. Selection of these assays as reference standards was based upon prevalence of use within the research community and/or frequency of selection of the assay target region as a qPCR target for the pathogen. As was the case for *W*. *bancrofti* detection, all *P*. *falciparum* reactions were performed in duplicate, using optimized and/or published reaction conditions. Building on previously published conventions [29], for all testing, a sample was considered positive if both replicate reactions produced Cq values ≤ 40. In the event that only one replicate reaction produced a positive result, the sample was re-tested in duplicate, and positivity was confirmed if at least one re-test replicate produced a positive result with a Cq value ≤ 40. Upon retesting, if neither replicate amplified with a Cq value ≤ 40, the sample was considered negative. In all cases of retesting, only retest results were reported. Of note, while technicians were not blinded to results summaries for comparator assays during sample processing, comparative individual sample results were not made available during the time of testing.

## Results

### Next generation sequencing, repeat analysis, and target selection

Illumina sequencing of *P*. *falciparum* produced 4.3 million paired-end reads of which 86.4% passed the filter for read quality with a quality (Q) score ≥ 35 (inferred base call accuracy of 99.95%). *W*. *bancrofti* paired-end sequencing produced 15 million reads, of which 83.4% passed filter with a Q score ≥ 35. For each library, 500,000 reads of uniform length (75bp for *Wb* and 100bp for *Pf)* were then randomly selected for analysis using RepeatExplorer2 and/or TAREAN, allowing for the generation of clusters and the identification of consensus sequences. For both pathogens, highly represented clusters were selected based upon the number of component reads in conjunction with the length of the repeat sequence. Selected repeat clusters were estimated to represent 0.320% and 1.300% of the *W*. *bancrofti* and *P*. *falciparum* read totals respectively. Utilizing the consensus sequences for each selected cluster, PrimerQuest Software was next used to select primer-probe pairings for use in the development of a TaqMan-based qPCR assay for each target (Table 2). A Primer-BLAST analysis then provided preliminary *in silico* evidence that each assay would amplify DNA only from its intended target species.

**Table 2:** Primer and probe sequences for the newly described qPCR assays targeting the greatest copy-number tandem repeats.

Target Species	Forward Primer (5’→3’)	Reverse Primer (5’→3’)	Probe (5’→3’)
*Wuchereria bancrofti*	GCTGAAAAACATTCGCTTTTGAATG	GGGTAATTAAACCGGTGATCCT	ACAACAACTATATGGGAATGGTGCAGGT
*Plasmodium falciparum*	GTTACCATAAGACCTATATGAATGAAAG	GGTCTTAAATTGAGTAACTAAGATCA	ACGTAGGTCTTACATTAACTAACTCAGGTC

### Assay design

#### Assay optimization

Utilizing a matrix of doubling dilutions, primers were titrated to determine optimal concentrations for use in each assay. Based on resulting mean Cq values, final reaction concentrations for the new *P*. *falciparum* assay were determined to be 1 μM for both the forward and reverse primers (S1 Table). Optimal concentrations for the *W*. *bancrofti* assay were 125 nM for the forward primer and 1 μM for the reverse primer (S2 Table).

#### Assay specificity

To verify the species-level specificity of both newly developed assays, optimized primer/probe pairings were tested against a panel of parasite gDNA extracts, human gDNA, and DNA extracted from mosquitoes or human blood containing target parasites from various geographic locations. For both assays, amplification was only observed from reaction wells containing the appropriate target DNA. No off-target-amplification results were observed (Tables 3 and 4).

**Table 3:** Results of validation testing for the *Wb* TR1 assay.

Species	Source	Result
*Acanthocheilonema viteae*	Genomic DNA	-
*Brugia malayi*	Genomic DNA	-
*Brugia pahangi*	Genomic DNA	-
*Dirofilaria immitis*	Genomic DNA	-
*Loa loa*	Genomic DNA	-
*Onchocerca volvulus*	Genomic DNA	-
Human	Genomic DNA	-
*Wuchereria bancrofti* (American Samoa)	DNA Extract of Infected Mosquito	+
*Wuchereria bancrofti* (French Polynesia)	DNA Extract of Infected Mosquito	+
*Wuchereria bancrofti* (Ghana)	DNA Extract of Infected Mosquito	+
*Wuchereria bancrofti* (Haiti)	DNA Extract of Infected Mosquito	+
*Wuchereria bancrofti* (Sri Lanka)	DNA Extract of Infected Mosquito	+
*Wuchereria bancrofti* (Tuvalu)	DNA Extract of Infected Mosquito	+
*Wuchereria bancrofti* (Zanzibar)	DNA Extract of Infected Mosquito	+

**Table 4:** Results of validation testing for the *Pf* TR1 assay.

Species	Source	Result
*Acanthocheilonema viteae*	Genomic DNA	-
*Brugia malayi*	Genomic DNA	-
*Brugia pahangi*	Genomic DNA	-
*Dirofilaria immitis*	Genomic DNA	-
*Homo Sapiens*	Genomic DNA	-
*Loa loa*	Genomic DNA	-
*Onchocerca volvulus*	Genomic DNA	-
*Plasmodium cynomolgi*	Genomic DNA	-
*Plasmodium knowlesi*	Genomic DNA	-
*Plasmodium vivax*	DNA Extract of Infected Mosquito	-
*Plasmodium falciparum* (Strain 3D7)	Genomic DNA	+
*Plasmodium falciparum* (Strain 7G8)	Genomic DNA	+
*Plasmodium falciparum* (Strain HB3-B2)	Genomic DNA	+
*Plasmodium falciparum* (Strain D6)	Genomic DNA	+
*Plasmodium falciparum* (Strain DD2)	Genomic DNA	+
*Plasmodium falciparum* (Strain FCB)	Genomic DNA	+
*Plasmodium falciparum* (Strain FCR3CSA)	Genomic DNA	+
*Plasmodium falciparum* (Strain W2)	Genomic DNA	+

#### Reaction efficiency

Utilizing a titration of gDNA template, a reaction efficiency was calculated for each assay. Efficiencies were determined using the mean Cq results obtained for testing of template at masses of 2 ng, 200 pg, 20 pg, 2 pg, and 200 fg per reaction. Reaction efficiencies were determined using the qPCR library quantification tool available from the NEBioCalculator website (https://nebiocalculator.neb.com/#!/qPCRlibQnt) and can be found in Table 5.

**Table 5:** Reaction efficiencies for each assay utilized during experimental testing.

Assay	*Wb* TR1	*Wb* LDR	*Pf* TR1	*Pf* Ribosomal
**Efficiency**	98.44%	96.06%	89.91%	93.43%

#### Validation of genome copy number

Comparisons of Cq values across gDNA template concentrations produced average reductions in Cq value of 2.91 for the new *Wb* TR1 assay compared to the *Wb* LDR assay and 1.19 for the new *Pf* TR1 assay compared to the *Pf* Ribosomal assay (Table 6). While Cq value comparisons alone do not provide a meaningful measure of relative assay sensitivities, when similar reaction efficiencies exist, such values can provide useful information about genome target content, with reduced Cq values generally corresponding to increases in target copy number. Accordingly, these results provide evidence in support of the informatics-based output underlying our assay design.

**Table 6:** Comparison of mean Cq values for index and reference assays.

	*Wb* TR1 Mean Cq (±SD)	*Wb* LDR Mean Cq (±SD)	Difference in Means	*Pf* TR1 Mean Cq (±SD)	*Pf* Ribosomal Mean Cq (±SD)	Difference in Means
**1 ng**	13.81 (±0.14)	16.67 (±0.09)	2.86	17.76 (±0.06)	19.13 (±0.18)	1.37
**100 pg**	17.11 (±0.09)	19.92 (±0.03)	2.81	21.14 (±0.19)	22.51 (±0.10)	1.37
**10 pg**	20.41 (±0.08)	23.20 (±0.05)	2.79	24.61 (±0.06)	25.85 (±0.08)	1.25
**1 pg**	24.06 (±0.17)	26.95 (±0.18)	2.88	28.36 (±0.02)	29.23 (±0.19)	0.87
**100 fg**	27.10 (±0.31)	30.32 (±0.14)	3.22	32.06 (±0.36)	33.16 (±0.43)	1.11
**Mean**			**2.91**			**1.19**

#### Comparative analytical sensitivities

**Fig 1:**
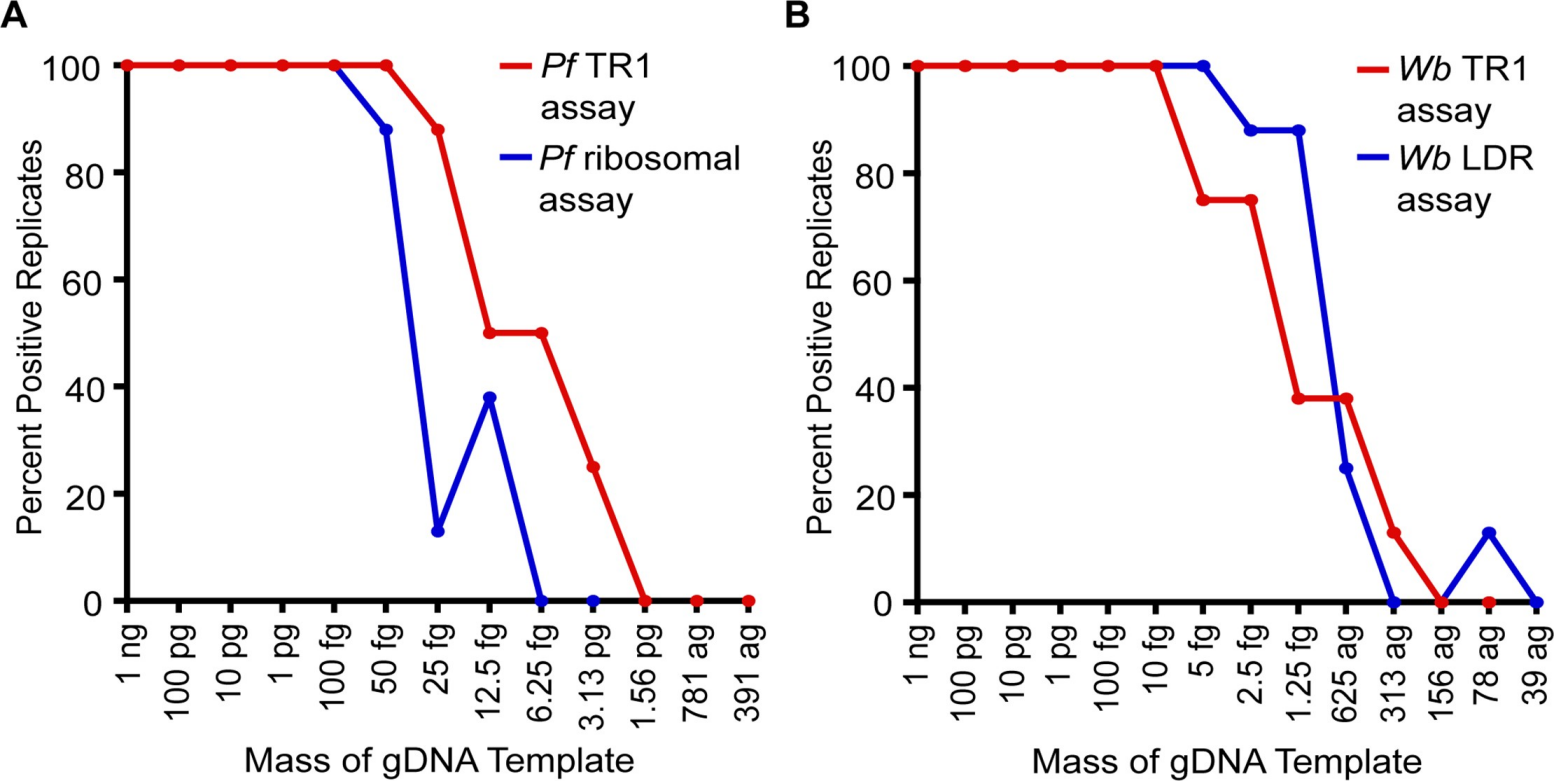
Analytical sensitivities of novel and published assays. Utilizing serial dilutions of gDNA template for each pathogen of interest, the final concentrations at which the novel (*Pf* TR1 and *Wb* TR1) and published (*Pf* ribosomal and *Wb* LDR) assays both consistently detected their target templates were identified. Utilizing these concentrations as starting points for each pathogen, a series of doubling dilutions was then created to further titrate the gDNA, and each assay was performed in octuplicate using each titrated standard in the second panel as template. Consistency of amplification for each assay was then plotted as a percentage for both *P*. *falciparum*-amplifying assays and both *W*. *bancrofti*-amplifying assays.

To investigate the comparative analytical sensitivities of our assays, a series of doubling dilutions of gDNA template were tested comparing the *Wb* TR1 and *Wb* LDR assays and the *Pf* TR1 and *Pf* Ribosomal assays. For both comparisons, the template dilution series began at the experimentally determined limit of consistent detection, meaning the final mass of template at which both assays being compared allowed for consistent detection of pathogen signal upon testing in octuplicate reactions. For detection of *P*. *falciparum*, this limit occurred at the 100 fg level. For detection of *W*. *bancrofti*, this limit was found to be 10 fg. Fig 1 reflects the percentage of replicate positivity for each assay as the input DNA was decreased. For both pathogens, the analytical sensitivity of each assay began to decline at comparable dilutions. When detecting *P*. *falciparum*, the ribosomal assay began to demonstrate inconsistent positivity at the 50 fg level while the new *Pf* TR1 assay was no longer consistent beginning at 25 fg (Fig 1A). For *W*. *bancrofti* detection, the *Wb* TR1 assay lacked consistency beginning at the 5 fg level, while the LDR-targeting assay remained consistent until the 2.5 fg level (Fig 1B).

#### Assay validation using field samples

To further evaluate comparative assay performance and to gauge field sensitivity and specificity, panels of mosquito DNA extracts were employed. Six hundred and sixteen extracts were tested utilizing both the *Pf* TR1 assay and *Pf* Ribosomal assay. This testing resulted in 179 samples which were positive utilizing the newly described *Pf* TR1 assay, but only 161 positive samples when testing with the ribosomal sequence-targeting assay. Of the *Pf* TR1 positive samples, 25 produced a negative result when tested with the *Pf* ribosomal assay, while only seven samples were positive using the ribosomal-targeting assay, but negative when tested using the *Pf* TR1 assay (Table 7). Recognizing any sample which tested positive by either assay to be a true positive, the sensitivity of the *Pf* TR1 assay was determined to be 96.24%, while the *P*. *falciparum* assay utilizing the ribosomal target had a sensitivity of 86.56%. In contrast, the *Wb* TR1 assay identified only a single positive sample that was determined to be negative utilizing the LDR assay, while testing with the LDR assay did not result in any positive samples that were undetected during *Wb* TR1 assay testing (Table 8).

**Table 7:** Comparative testing of field-caught mosquito DNA extracts using the *Pf* TR1 and *Pf* ribosomal assays.

	*Pf* Ribosomal Assay			
***Pf* TR1 Assay**		**Negative**	**Positive**	**Total**
	**Negative**	430	7	437
	**Positive**	25	154	179
	**Total**	455	161	616

**Table 8:** Comparative testing of field-caught mosquito DNA extracts using the *Wb* TR1 and *Wb* LDR assays.

	*Wb* LDR Assay			
***Wb* TR1 Assay**		**Negative**	**Positive**	**Total**
	**Negative**	265	0	265
	**Positive**	1	170	171
	**Total**	266	170	436

## Discussion

Given the wide-ranging global health impacts of both LF and malaria, the ability to accurately assess disease prevalence and burden is paramount. Though current DNA-based assays have vastly improved our ability to detect the causative agents of these diseases within a variety of sample types, the use of potentially sub-optimal targets can lead to both false-negative and false-positive results. Such results can negatively influence public health outcomes through their costly impacts on programmatic elimination efforts, improperly influencing decision-making processes and leading to economic losses.

With the help of next-generation sequencing, we have previously demonstrated that improvements in analytical and field sensitivity of qPCR-based diagnostic assays can be achieved through the identification and targeting of an organism’s tandemly repeat sequences of greatest copy number [24–26]. Similarly, a growing number of recent studies have demonstrated that the ability to accurately attribute infection to the proper causative agent is significantly impacted by the diagnostic target selected (reviewed in [26]). Understanding these challenges, and with a goal of improving existing tools, we have sought to identify and diagnostically exploit robust targets within the genomes of both *W*. *bancrofti* and *P*. *falciparum*. As reported above, these objectives were largely achieved with the development of the *Pf* TR1 assay, as both analytical and field sensitivities were improved. In contrast, improvements were much more modest when the *Wb* TR1 assay was compared against the commonly utilized *Wb* LDR assay for detection of *W*. *bancrofti*. While an analysis of comparative Cq values demonstrated the abundant nature of the *Wb* TR1 target, the resulting assay produced similar analytical and field sensitivities when compared with those obtained through testing with the commonly employed *Wb* LDR assay. The underlying causes for this unexpected similarity in sensitivities remain a mystery. One possibility could be that both assays were sufficient to allow for the detection of all, or nearly all true positive samples, resulting in their equivalent clinical performances. More surprising is the similarity of results seen during the testing of analytical sensitivity, as the reduced Cq values for *Wb* TR1 testing did not translate into improved limits of detection (Fig 1B). However, given that the analytical sensitivity for both assays remained equivalent until concentrations of template were reduced to levels less than 10 fg/μL, it is possible that this homogenization of results simply reflects the stochastic variation that is seen at these low levels of target. Given that a single microfilaria is estimated to contain approximately 100 pg of genomic DNA [30], these results again suggest that both the LDR reference assay and the the *Wb* TR1 assay are likely sufficiently sensitive to detect all true positive samples.

The results of our *W*. *bancrofti* repeat analysis present an interesting case. Previous analyses of various other multicellular eukaryotic pathogens using the RepeatExplorer-based pipeline have all suggested that the highest copy-number genomic elements are tandemly-repeated non-coding satellite sequences [24–26, 31]. In all cases, these repeat targets have significantly improved both the sensitivity and specificity of qPCR-based detection when compared to conventionally used targets such as ribosomal DNA. Unexpectedly, our analysis of the *W*. *bancrofti* genome identified a ribosomal repeat, rather than a satellite repeat, as the tandemly-repeated sequence element of putatively greatest copy number. Typically, such ribosomal targets raise concerns for potential cross-reactivity with closely related species due to the genetic conservation of these DNA elements. While analytical specificity testing of the *Wb* TR1 assay did not result in the false positive amplification of any related nematodes, testing against an all-inclusive set of genetically similar species is difficult and impractical. As such, there remains an increased risk for such cross-reactivity, and this risk should be considered when making decisions about the most appropriate assay for use in a given study. Despite the greater target copy number (as evidenced by comparative Cq values), improved confidence in specificity may render the LDR-targeting assay the best choice for many applications. This is particularly true considereing the similar analytical and clinical sensitivies seen during comparative testing.

While our results suggest improved analytical sensitivity and field-based detection of *P*. *falciparum* using the *Pf* TR1 assay, it is important to note that *P*. *falciparum* is not the leading cause of human malaria in WHO Region of the Americas, where *Plasmodium vivax* is most prevalent [32]. Similarly, *Plasmodium malariae* and *Plasmodium ovale* are significant agents of human disease. As such, the development of similar qPCR-based assays, with the capacity to sensitively and specifically identify these related pathogens should be developed. Such assays would be of use to global control and elimination efforts, while also aiding in accurate pathogen mapping, and furthering our understanding of species-specific responses to anti-malarial drugs.

While PCR-based diagnostics continue to play an increasingly important role in many neglected tropical disease control and elimination efforts, challenges pertaining to cost, infrastructure, and expertise remain significant. Fortunately, current developmental efforts are making PCR-based technologies increasingly accessible in resource limited settings, bridging the capacity gap and enabling the rapid deployment of the most reliable diagnostic methods to the areas where they are most urgently needed. As the global incidence of diseases such as LF and malaria continues to decrease, coordinated elimination efforts will increasingly rely upon optimal diagnostic options to confidently detect low levels of positivity, both within tested communities and within individual samples. Such detection will accurately inform stopping decisions facilitating the appropriate targeting of resources to where they are most urgently needed.

## Supporting information

S1 Checklist STARD checklist. Locations within the manuscript addressing each checklist item are indicated. This checklist provides the reader with criteria for assessing potential study biases. Additionally, criteria is intended to guide considerations regarding the generalizability of the results reported.
(DOCX)

S1 Table Results of primer optimization reactions for the *Wb* TR1 assay. Forward and reverse primers for the *Wb* TR1 assay were titrated and every possible pairing of titrated concentrations was tested. Results within the table are mean Cq values resulting from duplicate reactions.
(DOCX)

S2 Table Results of primer optimization reactions for the *Pf* TR1 assay. Forward and reverse primers for the *Pf* TR1 assay were titrated and every possible pairing of titrated concentrations was tested. Results within the table are mean Cq values resulting from duplicate reactions.
(DOCX)

S1 Flow diagram STARD flow diagram for the *Pf* TR1 assay. While the term “False” positive/negative is used, by convention, to represent discordant results when comparing index and reference assays, this designation more accurately represents disagreement only.
(DOC)

S2 Flow diagram STARD flow diagram for the *Wb* TR1 assay. While the term “False” positive/negative is used, by convention, to represent discordant results when comparing index and reference assays, this designation more accurately represents disagreement only.
(DOC)
