# Supplementary material for: Selection and exploitation of prevalent, tandemly repeated genomic targets for improved real-time PCR-based detection of Wuchereria bancrofti and Plasmodium falciparum in mosquitoes
Source: PLoS One. 2020 May 1;15(5):e0232325. doi: 10.1371/journal.pone.0232325 (PMC7194414; doi:10.1371/journal.pone.0232325)
Supplement: S1 Table — Forward and reverse primers for the Wb TR1 assay were titrated and every possible pairing of titrated concentrations was tested. Results within the table are mean Cq values resulting from duplicate reactions. (DOCX) [file pone.0232325.s002.docx]

**S1. Table**

|  | | Forward Primer (nM) | | | | |
| --- | --- | --- | --- | --- | --- | --- |
|  |  | **62.5** | **125** | **250** | **500** | **1000** |
| Reverse Primer (nM) | **62.5** | 26.05 | 21.50 | 19.55 | 19.32 | 19.07 |
|  | **125** | 24.28 | 19.96 | 18.27 | 17.51 | 17.72 |
|  | **250** | 23.12 | 19.46 | 17.67 | 17.29 | 17.10 |
|  | **500** | 22.86 | 19.17 | 17.68 | 17.11 | 17.01 |
|  | **1000** | 23.05 | 19.14 | 17.60 | 17.37 | 16.70 |
|  |  |  |  |  |  |  |
