# Supplementary material for: Selection and exploitation of prevalent, tandemly repeated genomic targets for improved real-time PCR-based detection of Wuchereria bancrofti and Plasmodium falciparum in mosquitoes
Source: PLoS One. 2020 May 1;15(5):e0232325. doi: 10.1371/journal.pone.0232325 (PMC7194414; doi:10.1371/journal.pone.0232325)
Supplement: S1 Flow diagram — While the term “False” positive/negative is used, by convention, to represent discordant results when comparing index and reference assays, this designation more accurately represents disagreement only. (DOC) [file pone.0232325.s004.doc]

# *Pf* TR1 Assay

n=616

*Pf* TR1 inconclusive

n=0

*Pf* TR1 negative

n=437

n=

*Pf* TR1 positive

n=179

## Reference standard

n=179

## Reference standard

n=437

## Reference standard

n=0

Agreement=430

“False” negative=7

Agreement=154

“False” positive=25

**S2 Flow diagram**. STARD flow diagram for a study of 616 DNA extracts isolated from pooled mosquitoes and tested using both the *Pf* TR1 (index) and *Pf* Ribosomal (reference) qPCR assays.
