# Supplementary material for: Selection and exploitation of prevalent, tandemly repeated genomic targets for improved real-time PCR-based detection of Wuchereria bancrofti and Plasmodium falciparum in mosquitoes
Source: PLoS One. 2020 May 1;15(5):e0232325. doi: 10.1371/journal.pone.0232325 (PMC7194414; doi:10.1371/journal.pone.0232325)
Supplement: S2 Flow diagram — While the term “False” positive/negative is used, by convention, to represent discordant results when comparing index and reference assays, this designation more accurately represents disagreement only. (DOC) [file pone.0232325.s005.doc]

# *Wb* TR1 Assay

n=436

*Wb* TR1 inconclusive

n=0

*Wb* TR1 negative

n=265

n=

*Wb* TR1 positive

n=171

## Reference standard

n=171

## Reference standard

n=265

## Reference standard

n=0

Agreement=265

“False” negative=0

Agreement=170

“False” positive=1

**S2 Flow diagram**. STARD flow diagram for a study of 436 DNA extracts isolated from pooled mosquitoes and tested using both the *Wb* TR1 (index) and LDR-1 (reference) qPCR assays.
